# Effect of TiO_2_ on Thermal, Mechanical, and Gas Separation Performances of Polyetherimide–Polyvinyl Acetate Blend Membranes

**DOI:** 10.3390/membranes13080734

**Published:** 2023-08-15

**Authors:** Khuram Maqsood, Asif Jamil, Anas Ahmed, Burhannudin Sutisna, Suzana Nunes, Mathias Ulbricht

**Affiliations:** 1 Department of Chemical Engineering, University of Jeddah, Jeddah 23890, Saudi Arabia; 2 Department of Chemical, Polymer and Composite Materials Engineering, University of Engineering and Technology (New Campus), Lahore 39021, Pakistan; 3 Department of Industrial and System Engineering, University of Jeddah, Jeddah 23890, Saudi Arabia; 4 Department of Food Engineering, Faculty of Industrial Technology, Bandung Institute of Technology, Jalan Let. Jen. Purn. Dr. (HC). Mashudi No.1, Sumedang 45363, Indonesia; 5 Biological and Environmental Science and Engineering Division (BESE), King Abdullah University of Science and Technology (KAUST), Thuwal 23955-6900, Saudi Arabia; 6 Lehrstuhl für Technische Chemie II, Universität Duisburg-Essen, 45117 Essen, Germany

**Keywords:** polyetherimide, polyvinyl acetate, titanium dioxide, blend membrane, composite membrane, gas separation

## Abstract

Blend membranes consisting of two polymer pairs improve gas separation, but compromise mechanical and thermal properties. To address this, incorporating titanium dioxide (TiO_2_) nanoparticles has been suggested, to enhance interactions between polymer phases. Therefore, the objective of this study was to investigate the impact of TiO_2_ as a filler on the thermal, surface mechanical, as well as gas separation properties of blend membranes. Blend polymeric membranes consisting of polyetherimide (PEI) and polyvinyl acetate (PVAc) with blend ratios of (99:1) and (98:2) were developed via a wet-phase inversion technique. In the latter, TiO_2_ was incorporated in ratios of 1 and 2 wt.% while maintaining a blend ratio of (98:2). TGA and DSC analyses were used to examine thermal properties, and nano-indentation tests were carried out to ascertain surface mechanical characteristics. On the other hand, a gas permeation set-up was used to determine gas separation performance. TGA tests showed that blend membranes containing TiO_2_ had better thermal characteristics. Indentation tests showed that TiO_2_-containing membranes exhibited greater surface hardness compared to other membranes. The results of gas permeation experiments showed that TiO_2_-containing membranes had better separation characteristics. PEI–PVAc blend membranes with 2 wt.% TiO_2_ as filler displayed superior separation performance for both gas pairs (CO_2_/CH_4_ and CO_2_/N_2_). The compatibility between the rubbery and glassy phases of blend membranes was improved as a result of the inclusion of TiO_2_, which further benefited their thermal, surface mechanical, and gas separation performances.

## 1. Introduction

Membrane technology resulting in benefits in terms of energy efficiency and from an economical point of view is regarded as a viable emerging technology in gas separation applications [1,2]. Nevertheless, due to the inherent trade-off between gas permeability and selectivity, commercially available membranes (either organic or inorganic) used in gas separation are available with particular constraints [3,4]. A probable and viable alternative in membrane-based gas separation technology is the use of polymer blends. However, one of the main challenges of this approach is establishing compatibility between paired polymers while maintaining the desired mechanical properties. Blending different polymers together can create composite materials that synergistically combine the properties of the individual components, which would otherwise be unattainable [5]. Adjusting the composition can obtain customized properties for specific applications. In particular, for membrane-based gas separations, it is advantageous to have a dense structure that facilitates the solution–diffusion mechanism. Therefore, in addition to considering the sizes of gases that pass through the membrane, it is also crucial to take into account the interaction between the molecules and the membrane.

Previous studies of polymer blends have highlighted the issue of immiscibility, which affects the physical and chemical properties of the product at the molecular level. Although immiscible blends are commercially available, their propensity to exhibit unstable phase morphology during melt processing frequently leads to inadequate mechanical performance [6,7]. To achieve a fine and stable morphology and improve the properties of the blend, the use of a suitable compatibilizer that acts as a surfactant has been explored [8]. This compatibilizer facilitates better interfacial interactions of the polymer phases and enhances the properties of the blend [8,9]. The most commonly used compatibilizer is a copolymer derived from monomers of the matrix polymer that is present in the blend. However, the complex synthesis process and limited applicability of specific blends have led researchers to seek better alternatives [10]. Recently, there has been a significant increase in studying the incorporation of nanoparticles into immiscible polymer blends. The nano-effects of these nanoparticles not only reduce phase separation behavior in the blend, but also enhance the mechanical and thermal stabilities of the resulting blend membrane [11]. Based on this hypothesis, incorporating nanoparticles into a PEI–PVAc blend can improve the physiochemical properties of the blend and enhance compatibility by refining the co-continuous structure of the blend through the addition of TiO_2_ nano-particles.

**Figure 1:**
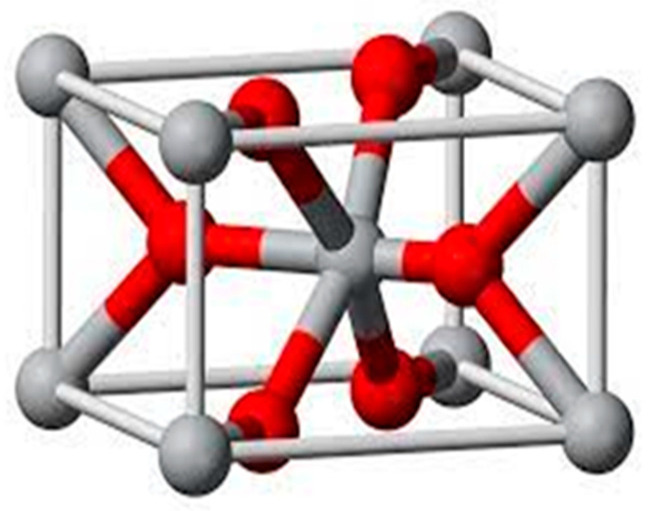
Structure of titanium dioxide.

Based on these observations as a reference point, this study aimed to investigate the impact of incorporating TiO_2_ into a PEI–PVAc blend membrane on its thermal, surface mechanical, and gas separation properties. The blending of a glassy and a rubbery polymer was intended to enhance the performance of the membrane systems by leveraging the distinctive gas separation characteristics of the parent polymers, while also introducing additional binding sites for carbon dioxide (CO_2_) at the molecular level. In the realm of membrane enhancement for gas separation, there are various additives, such as carbon black, carbon nanotubes, silica, nano-clay, and titanium oxide [12]. The structure of titanium dioxide is depicted in Figure 1. Li et al. reported improved mechanical stability in polymer membranes through the incorporation of TiO_2_ nanoparticles [13]. They also found that the addition of TiO_2_ reduced the solvent’s precipitation rate during membrane formation, leading to a denser skin layer in the cross-section of the membrane. Furthermore, with the addition of TiO_2_ nanoparticles, Madaeni et al. observed a corresponding rise in membrane wall thickness, as well as improved separation properties [14]. In a separate investigation, Ahmad et al. successfully prepared PVAc membranes incorporated with TiO_2_ nanocomposites, resulting in improved thermal stability and enhanced membrane performance [15]. Similarly, Cai et al. observed a reduction in the co-continuous phase of a polystyrene–polyamide (PS–PA6) blend upon the addition of TiO_2_ [16]. Hu et al. developed a poly(amide-imide)–TiO_2_ nanocomposite membrane that exhibited superior gas separation performance compared to pure poly(amide-imide), even at low TiO_2_ loading [17].

Based on these findings, this study hypothesized that incorporating inorganic moieties into the blend system would improve developed membranes’ mechanical and thermal characteristics. TiO_2_ was chosen as a key parameter for investigating the effects of incorporating these nanoparticles into the polymer blend, with the aim to improve the thermal and surface properties of blend membranes. Additionally, the gas separation performances of the membranes were analyzed by varying composition, temperature, and gas pressure.

## 2. Experimental

### 2.1. Materials

Prior to use, PEI pellets (with a melt flow index of approximately 9 g/10 min) were dehydrated in a 100 °C oven for 24 h to remove any moisture content. Conversely, PVAc beads (with a density of approximately 1.19 g/cm^3^ at 25 °C) were used as received without undergoing prior drying. An organic solvent, *N*-methyl-2-pyrrolidone (NMP), with a purity of 99.5%, was employed. In addition, particulate fillers were incorporated, consisting of TiO_2_ nanoparticles with a primary particle size of 21 nm. All the aforementioned chemicals were obtained from Sigma Aldrich (St. Louis, MO, USA).

### 2.2. Methods

In this study, different membranes, including pure PEI, PEI–PVAc blends, and PEI–PVAc blends with TiO_2_ nanoparticles, were prepared. Based on the optimized polymeric membrane ratio observed through previous investigations, the PEI–PVAc polymer blend’s ratio was established at (98:2) [18]. The pure and blend polymeric dope solutions were prepared following the methods outlined in our previous study.

However, for the PEI–PVAc–TiO_2_ membrane, a different approach was taken. In order to ensure homogeneity, TiO_2_ nanoparticles were first dispersed in NMP and stirred for 30 min. TiO_2_ particles were then subjected to an additional 30 min of ultrasonication in order to efficiently disperse them within the solvent. In a separate process, one half of the PEI was dissolved in NMP over the course of three hours at a temperature of 70 °C. Subsequently, to ensure complete dissolution, the rest of the PEI pellets and PVAc polymers were gradually added to the solution, while raising the temperature to 90 °C for a duration of nine hours. After the preparation of the solution, degassing procedures were conducted for a period of 12 h under ambient conditions to eliminate any trapped air bubbles.

### 2.3. Membrane Fabrication 

The membrane casting solution was applied onto a glass plate and uniformly spread using a casting knife by adjusting the film thickness to 150 µm. When the casting process was completed, the freshly formed membranes were placed in a bath of room temperature water. The membranes were then submerged in distilled water for three days, followed by air-drying at room temperature to ensure the complete removal of any remaining solvent. Table 1 provides the developed membranes and their respective properties.

**Figure 2:**
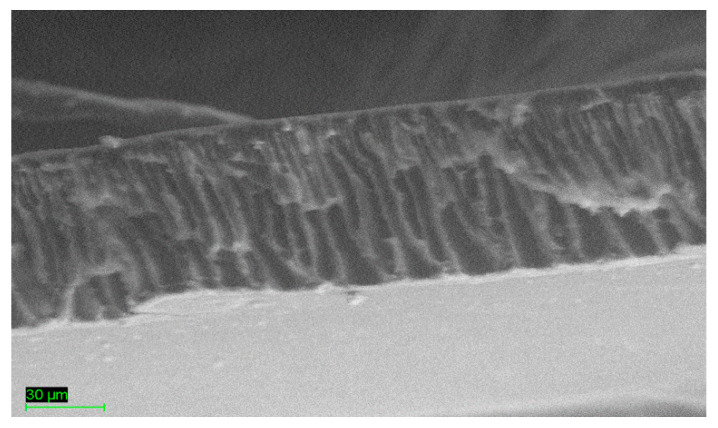
Cross-sectional view of the PEI–PVAc blend membrane.

The phase inversion method was adopted; as a result, the developed membrane was asymmetric, with a dense layer at the top and finger-like pores beneath, as depicted in Figure 2.

### 2.4. Characterization and Gas Permeation Tests of the Developed Membranes

#### 2.4.1. Thermal Analysis of the Membranes

Using a TGA (thermogravimetric analysis) analyzer (Perkin Elmer, Waltham, MA, USA, STA6000) in a nitrogen atmosphere, the blend membranes’ thermal characteristics were assessed. The measurements were carried out in the 30 to 600 °C temperature range with the heating rate set at 10 °C/min. The glass transition temperatures (*T_g_*) of the cast membranes were determined using DSC (differential scanning calorimetry) analysis. The membrane samples were heated in a nitrogen atmosphere from room temperature to 250 °C, and then returned to room temperature at a rate of 10 °C/min.

#### 2.4.2. Nano-Indentation Technique

**Figure 3:**
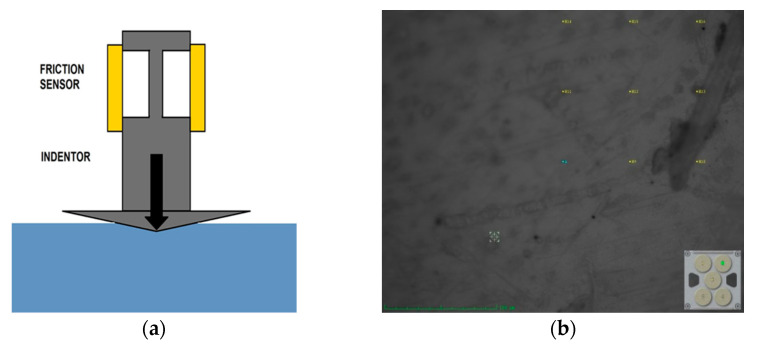
Schematic diagram of (**a**) indenter on the surface and (**b**) PEI membrane surface and indentation points.

Indentation tests were performed using the continuous stiffness measurement technique, which involves monitoring and recording the dynamic load and indenter of a diamond tip using the three-sided pyramid geometry displacement of a three-sided pyramidal diamond (Berkovich) indenter, as depicted in Figure 3a. The indenter had a tip radius of 0.219 µm with an effective tip-opening angle of 140.6°. This test was carried out in load-controlled mode, with the indentation load maintained at approximately 100 mN. At least 9 points were indented on each membrane; the specific indentation points for the pure PEI membrane sample are depicted in Figure 3b. Nanoindentation provided a means to measure mechanical properties of thin layers.

#### 2.4.3. Gas Permeation Study of the Developed Membranes

Permeability testing for pure CO_2_, N_2_, and CH_4_ gases was performed on developed membranes using a gas permeation unit specifically designed for these measurements. During testing, the gas pressure and permeation testing temperature were varied. To ensure accurate measurements, any remaining gases in the device were expelled using a vacuum pump prior to testing to ensure precise measurements. Maintaining atmospheric pressure kept the pressure on the permeate side of the membrane constant. The permeation rates of gas streams were measured using a bubble flow-meter capable of detecting flow rates as low as 100 mL/min. The following equation was used to calculate the gas permeation:

(1) $$\frac{P_{x}}{l}=\frac{Q}{A∆P}\frac{273.15}{T}\;$$

(2) $$\alpha_{xy}=\frac{P_{x}}{P_{y}}\;$$

The GPU measurement unit is used to quantify gas permeance (*P_x_/l*), where ‘*x*’ denotes the gas being studied (N_2_, CO_2_, or CH_4_). The volumetric flow rate is represented by ‘*Q*’, and the effective surface area of the membrane is denoted by ‘*A*’. The permeation process occurs at a specific temperature (*T*) and is conducted by applying a pressure difference (Δ*P*). The ideal selectivity, denoted as *α_xy_*, represents the ratio of the permeabilities of the competing incident gases x and y across the membrane.

## 3. Results and Discussion

### 3.1. Thermal Analysis

**Figure 4:**
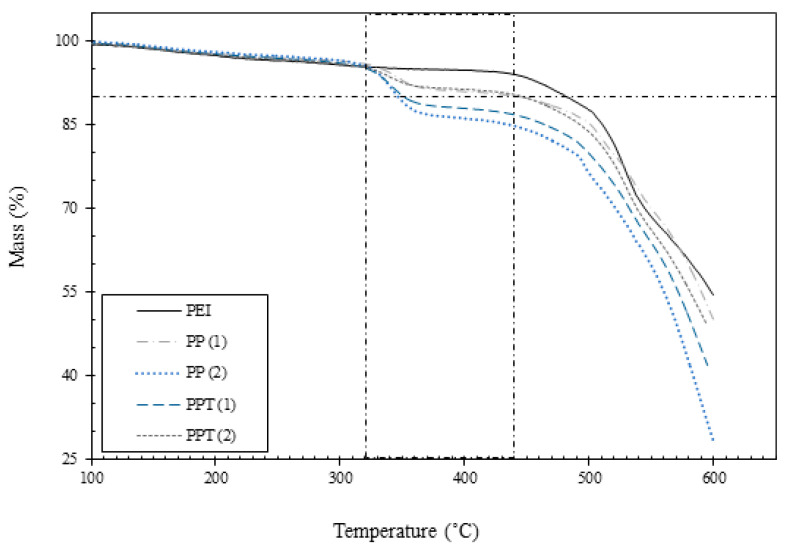
TGA analysis of developed membranes.

The thermal properties of the developed membranes were assessed through TGA and DSC analysis. Decomposition curves (Figure 4) indicated a single major mass loss for all membrane fibers. Notably, no weight loss was observed below 100 °C, indicating the absence of moisture in the developed membranes. The pure PEI membrane exhibited an onset temperature of approximately 440 °C, consistent with the previously published literature [19]. A 10% weight loss was used as a reference point to compare the degradation of the membranes. Weight loss between 100 and 300 °C is referred to as the mass of solvent retained in the membranes that evaporated as the temperature increased.

Furthermore, it was observed that the addition of PVAc to the PEI matrix resulted in a decrease in thermal properties as the proportion of the rubbery phase increased. In reference to the degradation temperature, neat PEI displayed a degradation temperature of 482 °C, which decreased to 340 °C for PP(2). These findings imply that adding rubbery PVAc to the glassy PEI matrix reduces its thermal properties. However, this trend was reversed with the addition of TiO_2_ into the blend membranes. The incorporation of 2 wt.% of TiO_2_ into the PEI–PVAc blend led to a recovery in the degradation temperature, which reached 450 °C. This indicates that an improvement in thermal stability was achieved by adding titanium to the blend membrane. Similar observations have been reported, such as the development of a polyethersulfone (PES) and PVAc blend membrane, which exhibited lower thermal stability compared to a plain PES membrane [20]. However, the addition of TiO_2_ reversed the thermal stability and restored it. These observations have also been reported in other studies [21].

The stiffness of polymeric materials is characterized by their *T_g_* values, which were determined through DSC analysis, and are shown in Table 1. It can be observed that the *T_g_* of pure PEI was approximately 212 °C, which aligns with the previous literature [18]. As the proportion of the rubbery PVAc component increased in the blend, the flexibility of the structure also increased, resulting in a lower *T_g_*, as indicated in Table 1. However, the presence of a single *T_g_* value suggests a uniform blending of the two polymers in the blend samples.

Nevertheless, the introduction of TiO_2_ resulted in a distinct and singular *T_g_* value across all tested membranes, indicating a molecular-level combination of polymer chains and TiO_2_, and the formation of a new material. The TiO_2_ content of blend membranes caused the *T_g_* value to increase. The homogeneous dispersion of titanium nanoparticles, which combined with the polymeric chains and decreased the free volume within the polymer structure, was responsible for this increase. Consequently, this restriction in the movement of chain fragments caused the polymer chains to become more rigid and raised the *T_g_* value. Numerous authors have extensively documented the rise of *T_g_* with the incorporation of TiO_2_ into the polymer matrix in the literature [22].

### 3.2. Surface Mechanical Analysis

**Figure 5:**
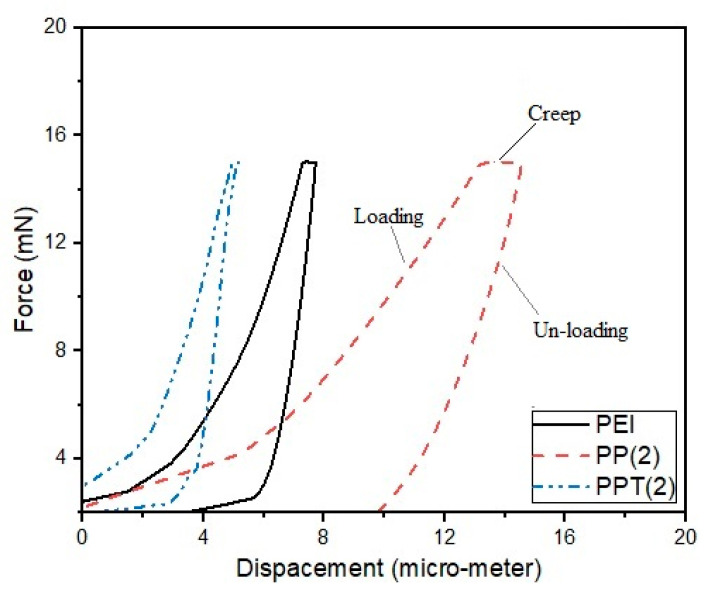
Load displacement analysis of casted membranes.

Figure 5 illustrates the compliance curve of neat PEI, PP(2), and PPT(2) membranes. A total of nine indents were applied to each sample, with a spacing of 40 μm between them. The membrane samples were subjected to a constant load of 15 mN, resulting in a maximum penetration depth of 15 μm. The unloading phase following the application of constant force exhibited a creep region, as depicted in Figure 5. Creep refers to a change in depth over time while maintaining a constant force, and it reached a new limit just before unloading. Subsequently, the indenter began to unload. The absence of overlap between the loading and unloading curves shown in Figure 5 suggests that the developed membranes demonstrated both elastic behavior and distinct plastic characteristics [23]. The loading and unloading curves appeared continuous and stable for all developed membranes.

The spacing between indents was adjusted to prevent overlapping of the internal stress generated around each indent. In the case of the neat PEI sample, the indenter penetrated up to a maximum contact depth of 7.7 μm, while this value increased to 14.00 μm for PP(2). However, the value decreased to 5.08 μm for the blend sample incorporating 2 wt.% of TiO_2_. It can be observed that the presence of PVAc in the PEI matrix reduced the hardness and stiffness of the surface. Conversely, incorporating TiO_2_ nanoparticles into the blend sample caused the surface to become stiff and hard. This can be attributed to the uniform dispersion of TiO_2_ nanoparticles within the polymer matrix, which imparted stiffness to the membranes. These findings are indirectly validated by the thermal analysis discussed in the previous section. Consistent with the previous literature, the addition of nanoparticles inside the polymer matrix resulted in a stiffer and harder surface [24]. Therefore, appropriate dispersion of nanoparticles can enhance membrane hardness and resistance to indenter penetration.

### 3.3. Gas Permeation Study

**Figure 6:**
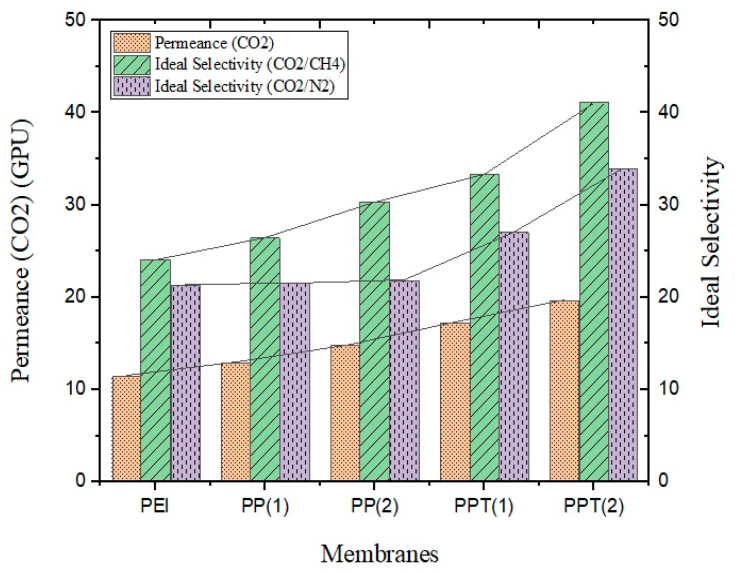
CO_2_ permeance and ideal selectivity (CO_2_/CH_4_, CO_2_/N_2_) of developed membranes.

Figure 6 depicts the CO_2_ permeance and ideal selectivity (CO_2_/CH_4_, CO_2_/N_2_) of pure PEI, PEI–PVAc blend, and PES–PVAc–TiO_2_ membranes. The figure clearly demonstrates that the composite membrane outperformed the other membranes in terms of performance. CO_2_ permeance showed an increasing trend with the addition of PVAc. The permeance of CO_2_ in the PEI–PVAc (98:2) blend membrane was 26.1% higher than that of the neat PEI membrane at ambient temperature and 2 bar pressure. The inclusion of the rubbery PVAc phase in the blend led to polymer chains being loosely packed, resulting in increased free volume. This loose structure facilitated the diffusion of smaller molecules through the blend matrix. As a result, the addition of the rubbery PVAc component enhanced the permeation of CO_2_, CH_4_, and N_2_. Furthermore, the CO_2_ benefited from higher solubility and a smaller kinetic diameter, which gave it an advantage in dissolving and diffusing through the blend membrane matrix. This advantage was reflected in the relative ideal selectivity of the developed blend membrane. Similar observations were reported by Farman et al. when PVAc was added to PES [25]. They observed (compared to neat PES) an almost double value of CO_2_ permeance and a 3.6 times increase in ideal (CO_2_/CH_4_) selectivity at 10 bar pressure.

On the other hand, the incorporation of TiO_2_ also had a positive impact on the CO_2_ permeance of the mixed matrix membrane. The CO_2_ permeance increased as the TiO_2_ content in the blend increased. For a 2 wt.% addition of TiO_2_ filler in the blend, the permeation was 36% higher compared to the neat sample. The incorporation of TiO_2_ into the PES–PVAc polymer blend resulted in nanoparticle aggregation and the creation of voids at the TiO_2_–polymer interface, thereby enhancing gas diffusion through the membrane. Similar observations were reported by Abdullah et al. when TiO_2_ nanoparticles were incorporated into PES–PVAc blend membranes [20]. They observed a 32.4% increase in CO_2_ permeance and a 65.9% surge in ideal (CO_2_/CH_4_) selectivity at 10 bar pressure and 25 °C. Thus, the addition of TiO_2_ nanoparticles proves advantageous for both gas selectivity and CO_2_ permeance in blend polymeric membranes. A similar behavior can be expected for CO_2_/N_2_ selectivity, as the kinematic diameter of N_2_ gas molecules is also smaller compared to CO_2_.

### 3.4. Effect of Feed Pressure

**Figure 7:**
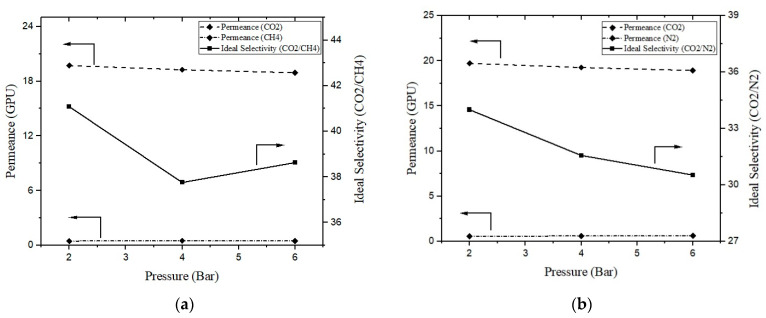
(**a**) CO_2_ permeance and ideal selectivity (CO_2_/CH_4_) and (**b**) CO_2_ permeance and (CO_2_/N_2_) selectivity of developed membranes against feed pressures.

Gas permeation tests were conducted at different feed pressures and at room temperature for the best membranes prepared by adopting a PEI–PVAc blend (98:2) and 2 wt.% TiO_2_ additions. Figure 7 depicts the gas permeations and ideal selectivity of the incident gases against the feed pressure; a noticeable trend can be observed wherein the permeance of CO_2_ gas generally decreased as the feed pressure increased. The decline observed in this study aligns with earlier findings that indicated a decrease in gas permeability for polarizable gases (such as CO_2_) with increased pressure in glassy polymers. These findings further support the dual-mode sorption hypothesis [26]. Additionally, increased feed pressure caused the membrane matrix to contract, which lessened the effect of swelling. Together, these two elements lessen CO_2_ permeability.

Nonetheless, as the membrane swelling decreases, the competitive transport between CH_4_, N_2_, and CO_2_ molecules is anticipated to become more pronounced. This phenomenon leads to a significant decline in gas selectivity. As depicted in Figure 7a, the ideal selectivity for CO_2_/CH_4_ gas decreased as the temperature increased. The value decreased from 41.1 to 38.6 as the temperature increased from 2 to 6 bar pressure. The effect of pressure change was more significant for CO_2_/N_2_ gas, as the selectivity decreased from 34 to 30.53. These results are consistent with those reported in the literature [27]. In another study, Matsuyama et al. developed polyethylenimine (PEI)-poly(vinyl alcohol) (PVA) blend membranes, and observed that with an increase in feed pressure, CO_2_ permeance decreased and N_2_ permeance remained almost the same, thereby decreasing CO_2_/N_2_ selectivity [28].

Therefore, it can be concluded that it is preferable to operate a prepared PPT(2) membrane at a relatively low feed pressure to attain a high separation performance for CO_2_ separation.

### 3.5. Effect of Temperature

**Figure 8:**
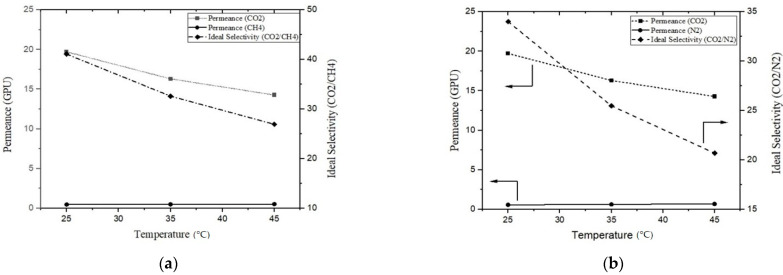
(**a**) CO_2_ permeance and ideal selectivity (CO_2_/CH_4_) and (**b**) CO_2_ permeance and (CO_2_/N_2_) selectivity of developed membranes against temperature.

For the most efficient PPT(2) membranes, gas permeation tests were carried out at different temperatures ranging from 25 to 45 °C and at 2 bar pressure. When the temperature increased from 25 to 45 °C, the CO_2_ permeance decreased significantly compared to other incident gases, such as CH_4_ and N_2_, as the solubility of CO_2_ gas’s polar nature is high compared to other gas molecules at room temperature. As the temperature increased, thermodynamically, the solubility of the CO_2_ gas significantly decreased compared to its counterparts, as depicted in Figure 8a,b. As the temperature increased, this declining trend in CO_2_ permeance had an impact on the developed membrane’s ideal selectivity. The ideal selectivity for CO_2_/CH_4_ over this temperature range decreased 25%. However, it was more prominent for CO_2_/N_2_, for which it reached 45%. These results suggest that these types of membranes are suitable at low to moderate temperatures. Khan et al. incorporated carbon nano sheets into a PEI matrix to develop composite membranes. The CO_2_/CH_4_ ideal selectivity of the developed membranes was tested at various temperatures from 25 to 50 °C; a slight decreasing trend was observed [29].

## 4. Conclusions

This study investigated the impact of incorporating TiO_2_ nanoparticles into a PEI–PVAc blend membrane on its thermal, surface mechanical, and gas separation properties. Incorporating TiO_2_ nanoparticles into the blend membrane enhanced its thermal stability, as evidenced by TGA analysis. This improvement was reflected in a higher observed *T_g_* value, as determined using DSC. Surface analysis revealed a stiffer and harder surface attributed to the uniform dispersion of nanoparticles within the polymer matrix compared to both neat and blend counterparts.

When compared to pure PEI, the PEI–PVAc blend membrane had better gas separation performance, and the addition of TiO_2_ nanoparticles improved ideal gas separation even further. Optimal results were obtained with a 2 wt.% TiO_2_ addition into the PEI–PVAc (98:2) matrix, achieving high selectivity for CO_2_/CH_4_ and CO_2_/N_2_. These findings demonstrate the potential of incorporating nanoparticles to improve the performance of polymer blend membranes in gas separation applications.

## Figures and Tables

**Table 1:** Compositions of developed membranes and their respective glass transition temperatures (Tg).

Membrane Code	PEI (wt.%)	PVAc (wt.%)	TiO_2_ (wt.%)	Tg (°C)
PEI	100	-	-	212
PP(1)	99	1	-	211
PP(2)	98	2		209
PPT(1)	98	2	1	210
PPT(2)	98	2	2	212

## Data Availability

Not applicable.
